# Antibody Responses in Cats Following Primary and Annual Vaccination against Feline Immunodeficiency Virus (FIV) with an Inactivated Whole-Virus Vaccine (Fel-O-Vax^®^ FIV)

**DOI:** 10.3390/v13030470

**Published:** 2021-03-12

**Authors:** Mark Westman, Dennis Yang, Jennifer Green, Jacqueline Norris, Richard Malik, Yasmin A. Parr, Mike McDonald, Margaret J. Hosie, Sue VandeWoude, Craig Miller

**Affiliations:** 1 Sydney School of Veterinary Science, The University of Sydney, Sydney, NSW 2006, Australia; dennis.cs.yang@gmail.com (D.Y.); j.green@sydney.edu.au (J.G.); jacqui.norris@sydney.edu.au (J.N.); 2 Centre for Veterinary Education, The University of Sydney, Sydney, NSW 2006, Australia; richard.malik@sydney.edu.au; 3 MRC, University of Glasgow Centre for Virus Research, The University of Glasgow, Glasgow G61 1QH, UK; Yasmin.Parr@glasgow.ac.uk (Y.A.P.); Margaret.Hosie@glasgow.ac.uk (M.J.H.); 4 Veterinary Diagnostic Services, The University of Glasgow, Glasgow G61 1QH, UK; Mike.McDonald@glasgow.ac.uk; 5 1619 Campus Delivery, College of Veterinary Medicine and Biomedical Sciences, Colorado State University, Fort Collins, CO 80523, USA; sue.vandewoude@colostate.edu; 6 College of Veterinary Medicine, Oklahoma State University, 250 McElroy Hall, Stillwater, OK 74078, USA; craig.miller@okstate.edu

**Keywords:** Australia, capsid protein, diagnosis, FIV, gp40, immunity, lentivirus, p24, transmembrane glycoprotein, veterinary science

## Abstract

Although the antibody response induced by primary vaccination with Fel-O-Vax^®^ FIV (three doses, 2–4 weeks apart) is well described, the antibody response induced by annual vaccination with Fel-O-Vax^®^ FIV (single dose every 12 months after primary vaccination) and how it compares to the primary antibody response has not been studied. Residual blood samples from a primary FIV vaccination study (*n* = 11), and blood samples from cats given an annual FIV vaccination (*n* = 10), were utilized. Samples from all 21 cats were tested with a commercially available PCR assay (FIV RealPCR^TM^), an anti-p24 microsphere immunoassay (MIA), an anti-FIV transmembrane (TM; gp40) peptide ELISA, and a range of commercially available point-of-care (PoC) FIV antibody kits. PCR testing confirmed all 21 cats to be FIV-uninfected for the duration of this study. Results from MIA and ELISA testing showed that both vaccination regimes induced significant antibody responses against p24 and gp40, and both anti-p24 and anti-gp40 antibodies were variably present 12 months after FIV vaccination. The magnitude of the antibody response against both p24 and gp40 was significantly higher in the primary FIV vaccination group than in the annual FIV vaccination group. The differences in prime versus recall post-vaccinal antibody levels correlated with FIV PoC kit performance. Two FIV PoC kits that detect antibodies against gp40, namely Witness^®^ and Anigen Rapid^®^, showed 100% specificity in cats recently administered an annual FIV vaccination, demonstrating that they can be used to accurately distinguish vaccination and infection in annually vaccinated cats. A third FIV PoC kit, SNAP^®^ Combo, had 0% specificity in annually FIV-vaccinated cats, and should not be used in any cat with a possible history of FIV vaccination. This study outlines the antibody response to inactivated Fel-O-Vax^®^ FIV whole-virus vaccine, and demonstrates how best to diagnose FIV infection in jurisdictions where FIV vaccination is practiced.

## 1. Introduction

Feline immunodeficiency virus (FIV) is a *Lentivirus* that infects domestic and feral cats (*Felis silvestris catus*) in addition to many non-domestic felids (e.g., lions, pumas and bobcats) and African hyenids [1,2]. Since its discovery in 1986 [3], FIV infection has been used as an animal model for human immunodeficiency virus (HIV-1) infection, especially in relation to the development of a HIV-1 vaccine [4,5,6,7,8]. As with FIV vaccination in cats, a critical consideration for the development and uptake of an effective HIV-1 vaccine will be the ability to quickly and accurately differentiate HIV-1-vaccinated and HIV-1-infected people using point-of-care (PoC) antibody test kits [9,10,11,12,13].

The culmination of a sustained FIV research effort was the release of a dual-subtype (FIV-A and FIV-D) formalin-inactivated whole-virus (IWV)/inactivated whole-cell (IWC) FIV vaccine (Fel-O-Vax^®^ FIV; Boehringer Ingelheim, Fort Dodge, IA, USA) in USA (2002), Canada (2003), Australia and New Zealand (2004), and Japan (2008) [14]. In all jurisdictions where it is still available (Australia, New Zealand and Japan), Fel-O-Vax^®^ FIV is licensed as requiring a primary course of three vaccinations, administered 2–4 weeks apart, followed by single re-vaccination every 12 months to ‘boost’ immunity. The release of this vaccine was initially met with resistance from some veterinarians since PoC test kits that could differentiate FIV-vaccinated and FIV-infected animals had not been identified, thereby creating a ‘diagnostic dilemma’ [15,16]. False-positive FIV test results in uninfected FIV-vaccinated cats have particularly serious implications in shelters where an incorrect FIV test result can lead to euthanasia [16,17]. Consequently, the World Small Animal Veterinary Association (WSAVA) Vaccination Guidelines in 2010 listed the Fel-O-Vax^®^ FIV vaccine as ‘Not Recommended’ [18].

A breakthrough in FIV diagnostics occurred when it was reported that two commercially available FIV PoC antibody test kits (Witness^®^, Zoetis Animal Health, Lyon, France; and Anigen Rapid^®^, BioNote, Gyeonggi-do, Korea) could accurately differentiate FIV-vaccinated and FIV-infected cats, using whole blood or saliva [11,19]. Similar findings were reported the following year using plasma as a diagnostic sample [20]. Additional testing determined that false-positive results with Witness^®^ and Anigen Rapid^®^ can occur in cats that have received a primary course of FIV vaccination within the preceding six months, and in these cats other discriminatory testing such as virus isolation should be performed [21]. A third FIV PoC antibody test kit (SNAP^®^ Combo, IDEXX Laboratories, Westbrook, ME, USA) does not differentiate vaccinated and infected cats, irrespective of how recently FIV vaccination has occurred, with false-positive results produced in more than 98% of vaccinated cats including some cats that had not been vaccinated for seven years [11,20]. The resolution of the FIV ‘diagnostic dilemma’ by using specific FIV PoC antibody kits played a role in the WSAVA Vaccination Guidelines re-classifying Fel-O-Vax^®^ FIV from ‘Not Recommended’ to ‘Non-Core’ in 2015 [22].

No studies have examined serial samples collected from cats after annual FIV vaccination to measure antibody response and evaluate the performance of FIV PoC antibody kits over time. The aim of the present study was to monitor the antibody response in cats recently administered an annual FIV vaccination, using both semi-quantitative FIV PoC test kits and quantitative laboratory assays, and to compare anti-FIV antibody responses induced by primary FIV vaccination vs. annual FIV vaccination.

## 2. Materials and Methods

### 2.1. Sample Populations

For the primary FIV vaccination cohort (*n* = 11), residual samples from client-owned kittens/cats were utilized [21]. Briefly, four kittens (<6 months of age) and seven cats (>6 months of age) were recruited, including six males and five females, ranging in age from three months to 7.6 years (median age 1.5 years; interquartile range [IQR] 0.5–2.6 years). Ten cats were non-pedigreed and one was a Ragdoll. All animals were neutered. For the present study, day 0 was considered the day that the kitten/cat presented to the veterinary clinic for the first dose of a primary course of Fel-O-Vax^®^ FIV vaccination.

For the annual FIV vaccination cohort (*n* = 10), client-owned cats were recruited including eight males and two females. The cats ranged in age from 1.3 years to 10.8 years (median age 6.0 years; IQR 2.6–8.9 years). All ten cats were non-pedigreed and neutered. Day 0 of this study was considered the day that the cat presented to the clinic for annual Fel-O-Vax^®^ FIV vaccination.

### 2.2. Vaccination

Fel-O-Vax^®^ FIV was administered subcutaneously into the dorsal interscapular space in all animals. None of the animals recruited for the primary vaccination study had been previously vaccinated against FIV. In accordance with the vaccine manufacturer’s guidelines, kittens/cats were vaccinated with Fel-O-Vax^®^ FIV on days 0, 28 and 56. All kittens/cats were kept with their owners for the duration of this study, and owners were encouraged to confine their cats indoors for two weeks after the final FIV vaccination to avoid possible FIV exposure prior to an immunological vaccination response occurring.

In the annual vaccination study, FIV vaccination histories for recruited cats were extracted from medical records and interrogated to ensure compliance with the vaccine manufacturer’s guidelines. Six of the ten cats had received their first primary FIV vaccination prior to six months of age and therefore did not have pre-vaccination FIV antibody testing performed; the remaining four cats received their first FIV vaccination at an age older than six months of age and had tested FIV-negative with a SNAP^®^ Combo PoC kit prior to FIV vaccination. All primary and annual FIV vaccinations had been administered in accordance with the vaccine manufacturer’s guidelines. The median number of annual FIV vaccinations that had been given prior to sampling was two (range 0–7, IQR 1–2.3). The median time since the last annual FIV vaccination was 362 days (range 319–396 days; IQR 324–372 days). All cats were kept with their owners for the duration of this study.

Cats in both the primary FIV and annual FIV vaccination groups were concurrently vaccinated with a core vaccine containing feline parvovirus virus (FPV), feline herpesvirus type-1 (FHV-1) and feline calicivirus (FCV) antigens (‘F3′), or FPV, FHV-1, FCV, feline leukemia virus (FeLV) and *Chlamydia felis* antigens (‘F5′). Core vaccines were administered subcutaneously into the dorsal interscapular space, a few centimeters away from the site of FIV vaccine administration, in all animals. F3 vaccines administered were either inactivated (i.e., adjuvanted; Fel-O-Vax^®^ 3; Boehringer Ingelheim, Fort Dodge, IA, USA) or modified-live formulations (Feligen^®^ RCP; Virbac Animal Health, Milperra, NSW, Australia), while the F5 vaccine administered is only available as an inactivated vaccine (Fel-O-Vax^®^ 5; Boehringer Ingelheim, Fort Dodge, IA, USA). The core vaccine dosing regime for kittens was three F3 vaccines administered one month apart, while the core vaccine dosing regime for adult cats was a single F3 or F5 annual booster dose. Supplementary Table S1 contains the details of core vaccines administered to each animal in the present study.

### 2.3. Determination of FIV Infection Status

Cats were tested for anti-FIV antibodies on day 0 using EDTA whole blood and commercially available PoC kits (primary FIV vaccination study—Witness^®^; Anigen Rapid^®^; SNAP^®^ Combo; and VETSCAN^®^ Rapid, Abaxis, Union City, CA, USA; annual FIV vaccination study—Witness^®^ and Anigen Rapid^®^). PCR testing was also performed on day 0 in both studies using a commercially available real-time PCR assay, and on the final day of sampling (day 238 for the primary vaccination study, day 42 for the annual vaccination study; FIV RealPCR^TM^, IDEXX Laboratories, East Brisbane, Queensland, Australia) (Table 1).

Witness^®^ and Anigen Rapid^®^ are immunochromatography test kits targeting envelope transmembrane glycoprotein gp40 for detection of FIV infection with sensitivities (Se) of 100%/100%, and specificities (Sp) of 98%/100%, respectively, under Australian conditions [11]. SNAP^®^ Combo is a lateral flow enzyme-linked immunosorbent assay (ELISA) kit that in Australia detects antibodies against matrix protein p15 and capsid protein p24, and in Europe detects antibodies against p15, p24 and gp40 [11]. In Australia, SNAP^®^ Combo had a Se/Sp of 100%/64% in a cohort that included 33% FIV-vaccinated cats [11]. In Europe, SNAP^®^ Combo had a Se/Sp of 100%/99.6% in a cohort that did not include any FIV-vaccinated cats [23]. VETSCAN^®^ Rapid is an immunochromatography kit that detects antibodies against p24 and has a reported Se/Sp in FIV-unvaccinated cats of 92%/99% [21,24]. FIV RealPCR^TM^ targets a conserved region of the *gag* gene and has a reported Se/Sp of 92%/99% in Australia [11]. Results from FIV RealPCR^TM^ testing are not affected by Fel-O-Vax^®^ FIV vaccination [11].

Cats were considered FIV-uninfected at the start of this study if they tested antibody negative with all PoC kits and PCR negative on day 0, and FIV-uninfected at the end of this study if they tested PCR negative at the final sampling (day 238 and day 42, respectively). All 21 cats were determined to be FIV-uninfected for the duration of this study.

### 2.4. Sampling Procedure

Kittens/cats recruited for the primary vaccination study were sampled fortnightly until four weeks after the third primary FIV vaccination (i.e., days 0, 14, 28, 42, 56, 70 and 84), and again six months later (day 238). Cats recruited for the annual vaccination study were sampled fortnightly for six weeks after vaccination (i.e., days 0, 14, 28 and 42) (Table 1). 

Sampling involved collecting approximately 1 mL of blood using jugular venipuncture and immediately transferring the specimen to an EDTA tube. When PCR testing was also performed, an additional 0.5 mL of blood was collected and aliquoted into a second EDTA tube. Blood tubes were stored at 4 °C until required. Testing for FIV antibodies with PoC kits was performed within 24 h of blood collection at the recruiting veterinary clinic or The University of Sydney, using whole blood from the EDTA tube and according to the manufacturer’s instructions (with one exception; see below). Following PoC testing, blood tubes were centrifuged for 3 min at 12,000 g, the plasma transferred to a plain tube using a sterile pipette, and the plasma specimens then stored at −80 °C until required. Plasma samples were transported at −20 °C to Colorado State University for anti-p24 microsphere immunoassay (MIA) testing, and at −80 °C to Veterinary Diagnostic Services (VDS), The University of Glasgow, for anti-gp40 ELISA testing. Samples from the annual vaccination study were also tested at VDS, Glasgow using European SNAP^®^ Combo PoC kits.

### 2.5. Evaluation of Antibodies to p24 Using a Microsphere Immunoassay (MIA)

Plasma samples from days 0, 14 *, and 28 ^†^ post-final vaccination (Primary: days 0, 70 * and 84 ^†^; Annual: days 0, 14 *, and 28 ^†^) were analyzed by microsphere immunoassay (MIA) for evaluation of antibodies against FIV capsid protein p24 (Table 1). Superscripts denote equivalent time points tested for primary vs. annual vaccination. Recombinant p24 protein used for MIA analysis was synthesized as previously described [25]. MIA was performed using previously established protocols involving conjugation of protein to carboxylated magnetic microspheres (MagPlex^®^ Microspheres, Luminex, Austin, TX, USA) [25,26,27]. Following conjugation, microsphere concentrations were determined by hemocytometer, and protein coupling confirmed by incubation of microspheres with phycoerythrin (PE)-conjugated detection antibodies [26]. Successful coupling was determined by a median fluorescence intensity (MFI) of >2000. Samples were then diluted 1:50 in assay buffer and incubated in duplicate with ~2500 conjugated beads per well, as described previously [26]. All samples were concurrently assayed with FIV-A (positive) and FIV-naïve (negative) reference samples diluted 1:50 in assay buffer, as well as four diluent control wells [26]. MFI was calculated from ≥100 microspheres per well (Bio-Plex™ Manager 5.0) and recorded as ‘negative fold’ (NF), where NF = (sample MFI)/(2 × negative control MFI). NF values were then used for statistical analyses [26,28]. All reagent concentrations, volumes, incubation times, positive and negative control reference samples, acceptable standard recovery, and data analysis were as described previously [25,26,27]. Samples from day 0 of the primary vaccination group served as FIV-unvaccinated controls. Samples were considered positive if NF values were greater than the mean NF of FIV-unvaccinated controls × 2 Standard Deviations (SD) [29]. Insufficient volume for MIA testing occurred in four primary vaccination samples and four annual vaccination samples.

### 2.6. Evaluation of Antibodies to gp40 Using a Laboratory ELISA

Plasma samples from days 0, 14 *, and 28 ^†^ post-final vaccination (Primary: days 0, 70 * and 84 ^†^; Annual: days 0, 14 *, and 28 ^†^) were analyzed by ELISA to detect antibodies binding the FIV transmembrane (TM; gp40) peptide as described previously [21] (Table 1). A nine-amino acid (AA) sequence peptide (CNQNQFFCK; cysteine–asparagine–glutamine–asparagine–glutamine–phenylalanine–phenylalanine–cysteine–lysine) from the highly conserved immunodominant TM2 domain of gp40 was used [30,31]. To the best of our knowledge, the Witness^®^ kit employs a 14 AA peptide, incorporating the nine AA sequence used for the laboratory-based TM ELISA, as its gp40 capture antigen [11,32]. The composition of the gp40 capture antigen used in the Anigen Rapid^®^ kit is unknown.

Known positive and negative controls were included on each test plate [21]. The positive control was pooled plasma from specific pathogen-free (SPF) cats experimentally infected with FIV, confirmed by Western blot and virus isolation. The negative control was a pooled sample from FIV-uninfected SPF cats, also confirmed by Western blot and virus isolation. Optical density (OD) results for samples were recorded as ‘negative fold’ (NF), where NF = (sample OD)/(2 × negative control OD). NF values were then used for statistical analyses [28]. Samples from day 0 of the primary vaccination group served as FIV-unvaccinated controls. Samples were considered positive if NF values were greater than the mean NF of FIV-unvaccinated controls ×2 SD [29]. Insufficient volume for ELISA testing occurred in four primary vaccination samples and four annual vaccination samples.

### 2.7. ‘Positive’ and ‘False-Positive’ Terminology for Antibody Test Results

Witness^®^ and Anigen Rapid^®^ are FIV antibody tests developed for the diagnosis of naturally acquired FIV infection by detection of antibodies to gp40. Therefore, a positive Witness^®^ or Anigen Rapid^®^ FIV result in a vaccinated/uninfected cat is reported as a ‘false-positive’ result. The MIA and ELISA testing performed was developed for the quantification of anti-p24 and anti-gp40 antibodies, not diagnosis of FIV infection. Therefore, a positive anti-p24 MIA or positive anti-gp40 ELISA result is reported as a ‘positive’ result.

### 2.8. Ethics Approval 

Animal ethics approval for both studies was granted by The University of Sydney (approval number 2015/858 for the primary vaccination study; approval number 2017/1167 for the annual vaccination study).

### 2.9. Statistical Analysis 

Statistical analyses were performed using the commercially available software: Genstat 18th Edition (VSN International, Hemel Hempstead, UK) and GraphPad Prism 9.0 (La Jolla, CA, USA). Signalment data of the two vaccination cohorts were compared by two-sample *t*-testing (age) and Fisher’s exact testing (sex and breed). Antibody levels (recorded as NF) within and between vaccination groups over time were compared by linear regression and repeated measures ANOVA using a mixed-effects model approach. Pearson’s correlation and logistic regression were used to compare anti-gp40 ELISA results to anti-gp40 PoC test results (i.e., Witness^®^ and Anigen Rapid^®^), and to analyze the anti-p24 and anti-gp40 antibody response following annual vaccination according to number of annual re-vaccinations received. For MIA (p24) and ELISA (gp40) testing, samples from two weeks after the third primary FIV dose were compared to samples from two weeks after annual FIV vaccination (i.e., day 70 * vs. day 14 *), and samples from four weeks after the third primary FIV dose were compared to samples from four weeks after annual FIV vaccination (i.e., day 84 ^†^ vs. day 28 ^†^). Statistical significance was considered when *p* < 0.05.

## 3. Results

### 3.1. Sample Populations

Kittens/cats recruited for the primary vaccination study were significantly younger than cats in the annual vaccination study (*p* = 0.005; two-sample *t*-test). There was no difference between vaccination groups in terms of sex and breed (*p* = 0.36 and 1.00, respectively; Fisher’s exact tests) (Table 2).

### 3.2. FIV Point-of-Care Testing (Primary Vaccination)

Results from FIV PoC testing in both vaccination groups are shown in Table 3. All four PoC kits tested false-positive at different points in the primary vaccination study. Witness^®^ and Anigen Rapid^®^ produced their highest rate of false-positive results on day 42 (two weeks after the second primary FIV vaccination; 64% and 55%, respectively), and were still false-positive in some cats on day 70 (two weeks after the final primary FIV vaccination; 55% and 36%, respectively) and day 84 (four weeks after the final primary FIV vaccination; 18% for both). Both kits tested negative in all cats on day 238. In total, 6/11 cats in the primary vaccination study tested false-positive with both Witness^®^ and Anigen Rapid^®^ at some time point, 3/11 never tested false-positive with either Witness^®^ or Anigen Rapid^®^, 1/11 tested false-positive only with Witness^®^ and not Anigen Rapid^®^ (day 14–day 70), and 1/11 tested false-positive only with Anigen Rapid^®^ and not Witness^®^ (day 42). SNAP^®^ Combo produced false-positive results in 100% of cats on day 28 (four weeks after the first primary FIV vaccination), and this result was reproduced for the duration of this study. VETSCAN^®^ Rapid produced false-positive results in 100% of cats between days 42 and 84, and 82% of cats on day 238.

### 3.3. FIV Point-of-Care Testing (Annual Vaccination)

Witness^®^ and Anigen Rapid^®^ produced negative results in all 10 cats for the duration of the annual vaccination study, including day 0. In contrast, 100% of cats in the annual vaccination study tested false-positive with SNAP^®^ Combo on day 0, and remained false-positive for the duration of this study (Table 3).

### 3.4. Antibodies to FIV p24 with MIA Testing

**Figure 1:**
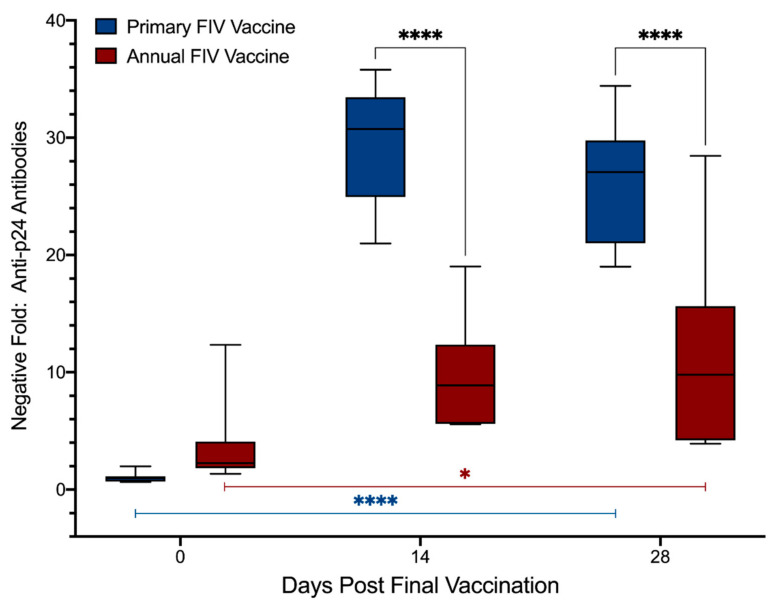
Anti-p24 antibody response from primary FIV vaccination was higher than annual FIV re-vaccination. Microsphere immunoassay (MIA) detected increases in anti-p24 antibodies over time in both primary and annual vaccination groups (Primary: *p* < 0.0001; Annual: *p* = 0.012; blue and red bars below the box plots, respectively; mixed-effects analysis). Anti-p24 antibodies differed significantly over time between groups (interaction, *p* < 0.0001) and at individual time points following vaccination (*p* < 0.0001, where the black bars joining the box plots indicate the analysis of the comparative time points), exhibited by increased antibody levels in primary vaccinated cats compared to annual vaccinated cats (mixed-effects analysis). Samples from days 0, 14 *, and 28 ^†^ post-final vaccination were compared (Primary: days 0, 70 * and 84 ^†^; Annual: days 0, 14 *, and 28 ^†^). Negative fold (NF) = (sample MFI)/(2 × negative control MFI). OD = optical density. * = *p* < 0.05, **** = *p* < 0.0001.

Results from MIA testing of plasma samples from days 0, 14 *, and 28 ^†^ after final vaccination (Primary: days 0, 70 * and 84 ^†^; Annual: days 0, 14 * and 28 ^†^) to detect anti-p24 antibodies are presented in Figure 1 and Supplementary Table S2. Compared to day 0, increased levels of anti-p24 antibodies were detected in both vaccination groups on days 14 * and 28 ^†^, indicating that both primary and annual FIV vaccination induced a significant anti-p24 antibody response (Primary: *p* < 0.0001, Annual: *p* = 0.012; mixed-effects analysis). The overall magnitude of the anti-p24 antibody response was 3.0× higher at day 14 * and 2.4× higher at day 28 ^†^ in the primary vaccination group compared to the annual vaccination group (treatment, *p* < 0.0001), as evidenced by increased levels of anti-p24 antibodies in primary vaccinated animals over time (interaction, *p* < 0.0001) and at individual time points (days 14 * and 28 ^†^: *p* < 0.0001) (mixed-effects analysis). Anti-p24 antibodies were detected in 6/10 cats in the annual vaccination group on day 0, indicating that antibodies persisted for at least 12 months after previous vaccination in a subset of cats (Table 4). Despite this finding, post hoc analysis revealed there was no significant difference between anti-p24 antibody levels in primary vs. annually vaccinated cats on day 0 (*p* = 0.56, Šídák’s multiple comparisons test).

### 3.5. Antibodies to FIV gp40 with ELISA Testing

**Figure 2:**
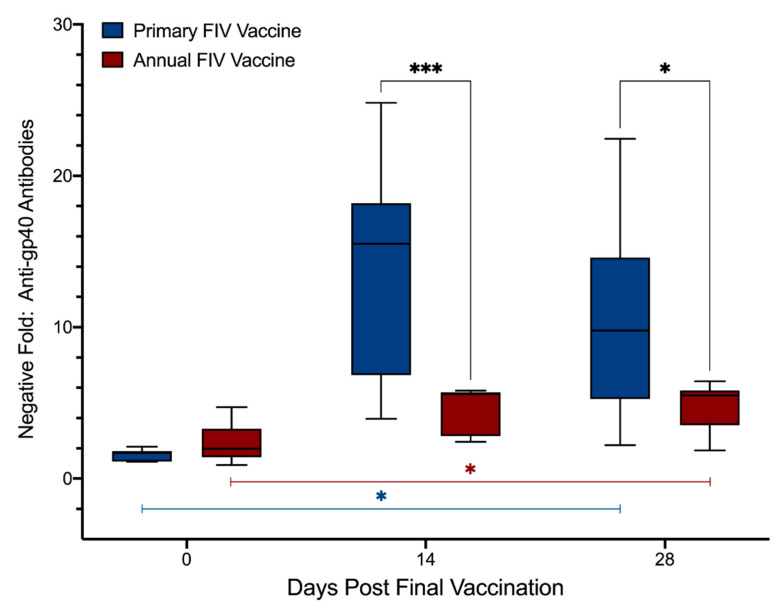
Primary FIV vaccination also induced a higher anti-gp40 antibody response than annual FIV re-vaccination. Anti-gp40 antibodies increased significantly over time in both primary and annual vaccination groups (Primary: *p* = 0.024; Annual: *p* = 0.0022; blue and red bars below the box plots, respectively; mixed-effects analysis). Anti-gp40 antibodies differed significantly over time between groups (interaction, *p* < 0.0001) and at individual time points following vaccination (day 14 *: *p* < 0.001; day 28 ^†^: *p* = 0.012, where the black bars joining the box plots indicate the analysis of the comparative time points), with primary vaccination inducing higher levels of antibodies compared to annual vaccination (mixed-effects analysis). Samples from days 0, 14 *, and 28 ^†^ post-final vaccination were compared (Primary: days 0, 70 * and 84 ^†^; Annual: days 0, 14 *, and 28 ^†^). NF = (sample OD)/(2 × negative control OD). OD = optical density. * = *p* < 0.05, *** = *p* < 0.001.

Results from ELISA testing of plasma samples from days 0, 14 *, and 28 ^†^ after final vaccination (Primary: days 0, 70 * and 84 ^†^; Annual: days 0, 14 * and 28 ^†^) to detect anti-gp40 antibodies are presented in Figure 2 and Supplementary Table S2. Similar to FIV p24, anti-gp40 antibody levels increased significantly over time in both primary and annual FIV vaccinated animals (Primary: *p* = 0.024; Annual: *p* = 0.0022), and the overall magnitude of the anti-gp40 antibody response in the primary vaccination group was 3.0× higher at day 14 * and 2.3× higher at day 28 ^†^ compared to the annual vaccination group (treatment, *p* < 0.0001) (mixed-effects analysis). Levels of anti-gp40 antibodies increased significantly in primary vaccinated cats over the course of this study (interaction, *p* < 0.0001) and at individual time points (day 14 *: *p* < 0.001; day 28 ^†^: *p* = 0.012) compared to the annual vaccinated animals (mixed-effects analysis). Anti-gp40 antibodies were detected by ELISA in 4/10 cats in the annual vaccination group on day 0, indicating that antibodies persisted for at least 12 months after previous vaccination in a subset of cats (Table 4), despite all 10 cats testing negative with Witness^®^ and Anigen Rapid^®^ PoC kits on day 0. Regardless of the persistence of anti-gp40 antibodies in some cats for 12 months, post hoc analysis revealed there was no significant difference between anti-gp40 antibody levels in primary vs. annually vaccinated cats on day 0 (*p* = 0.56, Šídák’s multiple comparisons test).

When anti-gp40 ELISA results from the primary FIV vaccination cohort were compared with results from the two anti-gp40 PoC kits (Witness^®^ and Anigen Rapid^®^), a significant correlation was observed (*p* < 0.0001), with some overlap (Supplementary Table S3). Specifically, increased levels of anti-gp40 antibodies in primary vaccinated cats were strongly associated with the likelihood of a false-positive PoC test result (Witness^®^ odds ratio [OR]: 1.46, *p* < 0.0001; Anigen Rapid^®^ OR: 1.25, *p* < 0.0001), such that 1 unit increase in anti-gp40 NF was associated with 46% increased likelihood of Witness^®^ testing false-positive and 25% increased likelihood of Anigen Rapid^®^ testing false-positive. Negative results with Witness^®^ had a range of NF values from 1.0 to 17.2 (median 2.9), while false-positive results with Witness^®^ ranged from 4.0 to 25.5 (median 17.3). Negative results with Anigen Rapid^®^ had a range of NF values from 1.0 to 20.5 (median 3.6), while false-positive results with Anigen Rapid^®^ ranged from 4.1 to 25.5 (median 16.6).

### 3.6. Comparing anti-p24 and anti-gp40 Antibody Responses to Number of Annual FIV Vaccinations

Multivariate logistic regression revealed a trend where the number of annual FIV vaccinations correlated with the detection of anti-p24 antibodies (*p* = 0.076) at 12 months post-vaccination (i.e., day 0 in the annual vaccination group) (Table 4).

Cats with higher numbers of annual FIV vaccinations were not more likely to test positive for anti-gp40-antibodies (*p* = 0.23; multivariate logistic regression) (Table 4).

## 4. Discussion

Results from the present study demonstrated that both vaccination protocols with Fel-O-Vax^®^ FIV–three primary vaccinations four weeks apart, and a single annual re-vaccination–induced a measurable vaccine-specific antibody response against FIV capsid protein p24 with a laboratory-based MIA, and against gp40 with a laboratory-based transmembrane peptide ELISA and PoC testing. The magnitude of antibody production over time was conspicuously greater in the primary FIV vaccination group compared to the annual FIV vaccination group for both antibodies assayed with laboratory-based tests. This difference between vaccination groups for anti-gp40 antibodies as measured by ELISA was consistent with results from Witness^®^ and Anigen Rapid^®^ FIV PoC testing [21], with some false-positive results seen in the primary FIV vaccination cohort from day 14 (two weeks after first primary FIV vaccination) until day 84 (four weeks after final primary FIV vaccination), but not in the annual FIV vaccination cohort. Consequently, Witness^®^ and Anigen Rapid^®^ kits are ideal for use in cats that have received an annual FIV re-vaccination within the preceding 12 months, irrespective of how recently the annual vaccination was given. 

SNAP^®^ Combo produced false-positive results in all cats from both the primary and annual vaccination groups, and therefore this kit cannot be used to distinguish FIV-vaccinated/FIV-uninfected cats from FIV-infected cats. If a positive FIV result is obtained with SNAP^®^ Combo in a cat with a possible history of FIV vaccination, follow-up testing (e.g., testing with Witness^®^, Anigen Rapid^®^, or the slightly less sensitive FIV RealPCR^TM^) should be pursued [11]. Since antibodies against FIV matrix protein p15 were not measured, we were unable to determine whether the false-positive results recorded with SNAP^®^ Combo were due to the detection of vaccine-induced anti-p15 antibodies, anti-p24 antibodies, or anti-gp40 antibodies (annual vaccination only). 

Using MIA and ELISA testing, antibodies against both p24 and gp40, respectively, were detectable on day 0 in a proportion of cats recruited for the annual vaccination study (6/10 and 4/10, respectively). This result demonstrated that anti-FIV antibodies induced by annual Fel-O-Vax^®^ FIV vaccination may persist for 12 months or more, consistent with previous reports [33,34,35]. Interestingly, in the present study only 2/6 cats that tested positive for anti-p24 antibodies on day 0 were also positive for anti-gp40 antibodies, suggesting that some cats had a stronger immunological response to vaccine p24 than vaccine gp40, or vice versa. Following a single annual FIV vaccination, antibody levels to both p24 and gp40 increased significantly over time, with peak antibody levels occurring 2–4 weeks after annual re-vaccination. Indeed, antibody levels were still increased at the final sampling (7/7 and 6/7 cats tested positive for anti-p24 and anti-gp40 antibodies, respectively, on day 42 with the laboratory-based assays; Supplementary Table S4). Although peak antibody levels induced by annual FIV vaccination were lower than following primary FIV vaccination, this finding does not necessarily suggest that vaccine-induced immunity from annual FIV vaccination is poorer than primary FIV vaccination. Humoral (antibody-mediated; Th2) immunity has been shown to be important for homologous FIV challenge, but cell-mediated (Th1) immunity is likely more critical for protection against FIV than humoral immunity, in particular for heterologous (e.g., field virus) challenge [1,7,14,15,36,37,38]. For example, one study reported that 84% of vaccinates were protected from heterologous FIV challenge 12 months after a primary course of Fel-O-Vax^®^ FIV vaccinations (three injections three weeks apart), despite anti-p24 antibody levels declining sharply by about three months following the third primary FIV vaccination [33]. 

Factors that may have contributed to the reduced humoral response against both p24 and gp40 following annual FIV re-vaccination compared to primary FIV vaccination include patient age, the number of FIV vaccines administered, and the re-vaccination interval. Cats in the annual vaccination group were significantly older than the cats in the primary vaccination group, and these older cats may have had a reduced antibody production potential. Flow cytometry studies have demonstrated an absolute reduction in T cells, B cells and natural killer cells in senior cats (10–14 years) compared to adult cats (2–5 years) [39]. Furthermore, an age-related gradual decline in relative percentage of lymphocytes has been observed, with analysis of the CD4+/CD8+ ratio defining two statistically distinct age groups (<8 months and >8 months of age) [40]. Future studies could examine the possible effect of age on immunological response to FIV vaccination by recruiting age-matched groups for primary and annual vaccination, and comparing the antibody responses between the groups by age. Such a study would likely need to be laboratory-based since administering a primary course of FIV vaccines to older cats is relatively uncommon in clinical practice in Australia, with primary vaccination usually occurring in kittens. Therefore, this was beyond the scope of the present field study. The number of FIV vaccines administered was likely also a factor, i.e., the ‘booster’ effect of three doses (primary vaccination) vs. one (annual vaccination). Peak antibody levels for both antibodies assayed in the primary vaccination group occurred two weeks after the second primary vaccination and maintained peak levels until two weeks after the third primary vaccination (i.e., day 42–day 70), demonstrating the booster effect of the second and third primary FIV vaccines (Supplementary Table S4). In elderly human subjects administered a single dose of an inactivated influenza vaccine, or two vaccine doses 12 weeks apart, booster vaccination yielded 14% higher post-vaccination titres than single vaccination [41]. Results of any vaccination regime is antigen and formulation dependent [42,43,44], and the immunological effects of changing the Fel-O-Vax^®^ FIV dosing schedule could be investigated. For example, trialing the administration of a second, or third, FIV dose as part of the annual re-vaccination schedule within a similar timeframe (i.e., 2–4 weeks apart), or reducing the annual re-vaccination interval (6 monthly rather than annually). The booster effect seen with multiple vaccines given within a short time frame likely relates to enhanced activation of the Th2 pathway, and as a result higher antibody titres, compared to the Th1-dominated response that occurs with a single dose program [45].

Finally, it is possible in the present study that residual anti-FIV antibodies in cats administered an annual vaccination neutralized some of the vaccine antigen, thereby effectively reducing the dose of vaccine antigen received, and therefore also reducing the magnitude of the antibody response induced by annual re-vaccination compared to primary vaccination. This has been shown to occur with FPV vaccination [46]. Similarly, a reduced antibody response following vaccination can result from the presence of maternally-derived antibodies (MDA). In kittens administered an inactivated vaccine against FHV-1 and FCV, vaccination was found to be more effective in the presence of low MDA than high MDA, as measured by virus neutralization to determine the proportion of kittens with protective antibody titres against FHV-1 and FCV [47]. In the present study, no difference in the magnitude of the anti-p24 or anti-gp40 antibody response was observed in the annual vaccination group according to the presence or absence of anti-p24 and anti-gp40 antibodies, respectively, on day 0 (Supplementary Table S5). For this reason, in future studies, it would be interesting to trial both shorter and longer re-vaccination intervals after the three-dose primary FIV vaccination schedule to determine the effect on post-vaccinal antibody response.

## 5. Conclusions

Despite the advent of molecular testing, detection of antibodies against FIV remains the most sensitive method for diagnosing FIV infection. Many FIV PoC antibody test kits are available around the world and they vary with regards to methodology (e.g., immunochromatography vs. ELISA), and choice of capture antigen used (e.g., p15, p24 and TM peptide), which in turn affects their accuracy. Differences in test kit performance are particularly pronounced in FIV-vaccinated cats. In countries where vaccination with Fel-O-Vax^®^ FIV is practiced, Witness^®^ and Anigen Rapid^®^ can be reliably used to diagnose FIV infection in cats administered a primary course of FIV vaccination more than six months prior to testing, and in any cats administered an annual FIV vaccination, irrespective of how recently it was administered. If FIV testing is required in a cat that has recently commenced or completed a primary course of FIV vaccination, a sensitive PCR assay should be used (e.g., FIV RealPCR^TM^), or if available, virus isolation. Investigation of the antibody response in cats vaccinated against FIV enhances our general understanding of vaccine-induced Th1 and Th2 responses, and differences that may occur depending on the length of the re-vaccination interval, which may be important for the development of other vaccines and dosing schedules.

## Figures and Tables

**Table 1:** Vaccination and testing schedule for primary and annual FIV vaccination studies. FIV antibody testing included testing with a variety of point-of-care (PoC) antibody kits, an anti-p24 antibody microsphere immunoassay (MIA), and an anti-gp40 antibody ELISA. PCR testing was performed by a commercial veterinary laboratory (FIV RealPCR^TM^, IDEXX Laboratories). For MIA and ELISA testing, samples from two weeks after the third primary FIV dose were compared to samples from two weeks after annual FIV vaccination (i.e., day 70 * vs. day 14 *), and samples from four weeks after the third primary FIV dose were compared to samples from four weeks after annual FIV vaccination (i.e., day 84 ^†^ vs. day 28 ^†^). Superscripts denote equivalent time points tested for primary vs. annual vaccination. Additional MIA and ELISA testing at other time points was performed (brackets) but not used for comparisons between vaccination groups; these results are included as Supplementary data.

	**Day 0**	**Day 14**	**Day 28**	**Day 42**	**Day 56**	Day 70 *	**Day 84 ^†^**	**Day 238**
**Primary vaccination study (*n* = 11)**								
FIV vaccination	×		×		×			
PoC antibody testing	×	×	×	×	×	×	×	×
MIA p24/ELISA gp40 testing	×	(×)	(×)	(×)	(×)	×	×	(×)
PCR testing	×							×
	**Day 0**	Day 14 *	**Day 28 ^†^**	**Day 42**				
**Annual vaccination study (*n* = 10)**								
FIV vaccination	×							
PoC antibody testing	×	×	×	×				
MIA p24/ELISA gp40 testing	×	×	×	(×)				
PCR testing	×			×				

**Table 2:** Signalment data of the two vaccination cohorts. All recruited animals were neutered. IQR = interquartile range, M = male, F = female, P = pedigreed, and NP = non-pedigreed. See Supplementary Table S1 for individual animal data.

Group	Age (Years)	Sex	Breed	*p* Value
Primary Vaccination	0.3–7.6 (median 1.5; IQR 0.5–2.6)	6M, 5F	1P, 10NP	*p* = 0.005 (age) *p* = 0.36 (sex) *p* = 1.00 (breed)
Annual Vaccination	1.3–10.8 (median 6.0; IQR 2.6–8.9)	8M, 2F	10NP	

**Table 3:** Cats in the annual FIV vaccine study did not elicit positive results on Witness^®^ or Anigen Rapid^®^ point-of-care (PoC) test kits. Primary vaccination table results have been taken from Westman et al. (2017) [21]. All PoC testing was performed using whole blood, except for testing with SNAP^®^ Combo in the annual vaccination study which was performed using plasma. There was insufficient plasma from ten samples in the annual vaccination study to perform FIV testing with SNAP^®^ Combo. VACC = vaccination with Fel-O-Vax^®^ FIV. NP = not performed. The numerator indicates the number of false-positive test results.

	**Day 0**	**Day 14**	**Day 28**	**Day 42**	**Day 56**	**Day 70**	**Day 84**	**Day 238**
**Primary vaccination study (*n* = 11)**	VACC		VACC		VACC			
Witness^®^	0/11	2/11	6/11	7/11	6/11	6/11	2/11	0/11
Anigen Rapid^®^	0/11	0/11	1/11	6/11	5/11	4/11	2/11	0/11
SNAP^®^ Combo	0/11	7/11	11/11	11/11	11/11	11/11	11/11	11/11
VETSCAN^®^ Rapid	0/11	0/11	3/11	11/11	11/11	11/11	11/11	9/11
PCR testing (FIV RealPCR^TM^)	0/11							0/11
	**Day 0**	**Day 14**	**Day 28**	**Day 42**				
**Annual vaccination study (*n* = 10)**	VACC							
Witness^®^	0/10	0/10	0/10	0/10				
Anigen Rapid^®^	0/10	0/10	0/10	0/10				
SNAP^®^ Combo	10/10	7/7	6/6	7/7				
VETSCAN^®^ Rapid	NP	NP	NP	NP				
PCR testing (FIV RealPCR^TM^)	0/10			0/10				

**Table 4:** Cats that received a higher number of annual FIV re-vaccinations were more likely to have antibodies against p24 12 months post-vaccination. In total, 6/10 and 4/10 cats in the annual vaccination group tested positive on day 0 for antibodies against p24 and gp40, respectively, demonstrating that these cats had a duration of antibody response of at least 12 months since their last FIV vaccination. Cats administered a higher number of annual FIV vaccinations were more likely to test positive for anti-p24 antibodies at 12 months post-vaccination (*p* = 0.076; multivariate logistic regression). There was no trend observed with anti-gp40 antibody ELISA results at 12 months in cats administered a higher number of annual FIV vaccinations (*p* = 0.23; multivariate logistic regression). Samples were considered antibody positive if NF values were greater than the mean NF of FIV-unvaccinated controls × 2 standard deviations. NF = negative fold, MIA = microsphere immunoassay testing, and ELISA = laboratory-based anti-FIV transmembrane peptide ELISA testing.

Anti-p24 Antibodies (MIA) 12 Months Post-Vaccination	Anti-gp40 Antibodies (ELISA) 12 Months Post-Vaccination	No. of Annual FIV Vaccinations (Prior to Enrollment)
−	−	0
−	+	1
+	−	1
−	−	2
+	−	2
+	−	2
−	+	2
+	+	2
+	−	3
+	+	7
6/10 cats	4/10 cats	

## Data Availability

All data presented in this paper are available on request.

## Supplementary Materials

The following are available online at https://www.mdpi.com/1999-4915/13/3/470/s1, Table S1: Individual animal signalment data, Table S2: Individual animal anti-p24 and anti-gp40 antibody results (day 0, day 14 and day 28 post-final vaccination), Table S3: Anti-gp40 antibody ELISA results arranged by PoC test result, Table S4: Individual animal anti-p24 and anti-gp40 antibody results (all time points tested), Table S5: Comparing antibody responses in the annual vaccination group according to the anti-p24/anti-gp40 antibody result on day 0.
